# Two subgroups in systemic lupus erythematosus with features of antiphospholipid or Sjögren’s syndrome differ in molecular signatures and treatment perspectives

**DOI:** 10.1186/s13075-019-1836-8

**Published:** 2019-02-18

**Authors:** Helena Idborg, Arash Zandian, Ann-Sofi Sandberg, Bo Nilsson, Kerstin Elvin, Lennart Truedsson, Azita Sohrabian, Johan Rönnelid, John Mo, Giorgia Grosso, Marika Kvarnström, Iva Gunnarsson, Janne Lehtiö, Peter Nilsson, Elisabet Svenungsson, Per-Johan Jakobsson

**Affiliations:** 1 Division of Rheumatology, Department of Medicine Solna, Karolinska Institutet, Karolinska University Hospital, 171 76 Stockholm, Sweden; 2 Division of Affinity Proteomics, SciLifeLab, Department of Protein Science, KTH Royal Institute of Technology, Stockholm, Sweden; 3 Clinical Proteomics Mass Spectrometry, Department of Oncology-Pathology, Science for Life Laboratory and Karolinska Institutet, Stockholm, Sweden; 4 Department of Immunology, Genetics and Pathology, Uppsala University, Uppsala, Sweden; 5 Unit of Clinical Immunology, Department of Clinical Immunology and Transfusion Medicine, Karolinska University Hospital, Karolinska Institutet, Solna, Stockholm, Sweden; 6 Section of Microbiology, Immunology and Glycobiology, Department of Laboratory Medicine, Lund University, Lund, Sweden; 7 Patient Safety Respiratory, Inflammation, Autoimmunity, Infection and Vaccines, AstraZeneca R&D, Gothenburg, Sweden

**Keywords:** Systemic lupus erythematosus, Antiphospholipid syndrome, Sjögren’s syndrome, Personalized medicine, Affinity-based proteomics, Subgroups

## Abstract

**Background:**

Previous studies and own clinical observations of patients with systemic lupus erythematosus (SLE) suggest that SLE harbors distinct immunophenotypes. This heterogeneity might result in differences in response to treatment in different subgroups and obstruct clinical trials. Our aim was to understand how SLE subgroups may differ regarding underlying pathophysiology and characteristic biomarkers.

**Methods:**

In a cross-sectional study, including 378 well-characterized SLE patients and 316 individually matched population controls, we defined subgroups based on the patients’ autoantibody profile at inclusion. We selected a core of an antiphospholipid syndrome-like SLE (aPL+ group; positive in the lupus anticoagulant (LA) test and negative for all three of SSA (Ro52 and Ro60) and SSB antibodies) and a Sjögren’s syndrome-like SLE (SSA/SSB+ group; positive for all three of SSA (Ro52 and Ro60) and SSB antibodies but negative in the LA test). We applied affinity-based proteomics, targeting 281 proteins, together with well-established clinical biomarkers and complementary immunoassays to explore the difference between the two predefined SLE subgroups.

**Results:**

The aPL+ group comprised 66 and the SSA/SSB+ group 63 patients. The protein with the highest prediction power (receiver operating characteristic (ROC) area under the curve = 0.89) for separating the aPL+ and SSA/SSB+ SLE subgroups was integrin beta-1 (ITGB1), with higher levels present in the SSA/SSB+ subgroup. Proteins with the lowest *p* values comparing the two SLE subgroups were ITGB1, SLC13A3, and CERS5. These three proteins, rheumatoid factor, and immunoglobulin G (IgG) were all increased in the SSA/SSB+ subgroup. This subgroup was also characterized by a possible activation of the interferon system as measured by high KRT7, TYK2, and ETV7 in plasma. In the aPL+ subgroup, complement activation was more pronounced together with several biomarkers associated with systemic inflammation (fibrinogen, α-1 antitrypsin, neutrophils, and triglycerides).

**Conclusions:**

Our observations indicate underlying pathogenic differences between the SSA/SSB+ and the aPL+ SLE subgroups, suggesting that the SSA/SSB+ subgroup may benefit from IFN-blocking therapies while the aPL+ subgroup is more likely to have an effect from drugs targeting the complement system. Stratifying SLE patients based on an autoantibody profile could be a way forward to understand underlying pathophysiology and to improve selection of patients for clinical trials of targeted treatments.

**Electronic supplementary material:**

The online version of this article (10.1186/s13075-019-1836-8) contains supplementary material, which is available to authorized users.

## Background

Systemic lupus erythematosus (SLE) is an autoimmune disease with a heterogeneous presentation covering a wide range of phenotypes, from subtle symptoms to life-threatening conditions. The heterogeneous presentation of SLE is a major obstacle in clinical trials as there are no good biomarkers to measure disease activity in general or to compare disease activity in different organ systems. Response to treatment may also differ between subgroups [1, 2]. Due to this heterogeneity, treatments with good effect in SLE subgroups will likely fail to show efficacy in SLE overall. Consequently, poorly delineated and unrecognized SLE subgroups may blur important outcomes of clinical trials and thus prevent subgroups of patients from achieving improved quality of life. The lack of biomarkers also hampers accurate diagnosis, prediction of prognosis and treatment [3].

The diagnostic overlaps between SLE, anti-phospholipid syndrome (APS), and Sjögren’s syndrome (SS) are evident in the clinic. It is in this context important to remember that the present APS criteria advice against separating APS into primary and secondary subsets, since there is no evidence that the clinical consequences of anti-phospholipids (aPL) among patients in these two categories differ [4]. Vascular events, main characteristics of APS, have consistently been associated with aPL in several prospective SLE studies [5–7]. Cluster analyses based on autoantibody profile have been performed to detect subgroups of SLE patients. To et al. reported three major clusters consisting of an aPL, a Sjögren’s syndrome antigen A/B (SSA/SSB, anti-Ro/La), and an anti-Smith/ribonuclear (Sm/RNP) autoantibody cluster in a large American SLE cohort [8]. Similar antibody clusters were identified in a Turkish SLE cohort: an aPL, a SSA/SSB, and a Sm/RNP cluster, but they also identified an anti-double stranded DNA (anti-dsDNA) antibody cluster [9]. The aPL linked clusters were associated with higher damage scores according to the Systemic Lupus International Collaborating Clinics (SLICC) damage index (SDI) [9], in particular with vascular damage [8], i.e., the main reason for a shorter life expectancy in SLE [7]. To prevent the occurrence of vascular and permanent damage, it is therefore very important to identify patients belonging to the aPL+ SLE cluster early.

Based on own clinical observations and inspired by previous studies [8, 9], we defined two subgroups: a SSA/SSB+ and an aPL+ subgroup. In order to get a straightforward and simply applicable definition of the second subgroup, we decided to use lupus anticoagulant (LA), a strong and well-recognized APS predictor [4, 10]. We combined data from affinity-based proteomics, routine clinical measurements, and biochemical assays to investigate possible differences between the two subgroups. We hypothesized that there are important molecular/pathogenic differences underlying the aPL+ and SSA/SSB+ SLE sub-phenotypes.

## Methods

### SLE cohort

When this study was initiated, the Karolinska SLE cohort comprised 378 consecutive SLE patients and 316 age- and gender-matched population-based controls. All SLE patients fulfilled at least four of the revised American College of Rheumatology classification criteria for SLE [11]. At inclusion, all participants were subject to a structured clinical examination and an extensive protocol was filled out. Disease activity was determined by both the Systemic Lupus Activity Measure (SLAM) of global lupus activity and by Systemic Lupus Erythematosus Disease Activity Index 2000 (SLEDAI-2K) [12]. Organ damage was scored using the SDI [9]. EDTA plasma was collected from fasting patients and controls and stored at − 70°C. The ethical board at the Karolinska University Hospital approved the study (reference number 03-556). All study participants gave written informed consent to participate.

#### Definition of SLE subgroups

We were interested in the cluster with the most severe clinical picture, i.e., the aPL-positive cluster, and the largest cluster, i.e., the anti-SSA/SSB+ cluster. To capture a representative core of these two clusters, we used a strict autoantibody-based definition:

- The *aPL+ subgroup* was defined as SLE patients who were positive in the lupus anticoagulant (LA) test and negative for all three SSA (Ro52 and Ro60) and SSB antibodies.
- The *SSA/SSB+ subgroup* was defined as patients who were positive for all three of SSA (Ro52 and Ro60) and SSB antibodies but negative in the LA test.

Positivity/negativity was based on analyses of samples taken at inclusion.

### Biochemical assays

The immunological profile was determined in all patients by established and standardized techniques at the laboratories of clinical immunology and clinical chemistry at Karolinska University Hospital, as previously described [5]: e.g., antibodies to specific nuclear antigens (dsDNA, SSA-Ro52, SSA-Ro60, SSB, Sm) and phospholipids (cardiolipin immunoglobulin (Ig) G/IgM and β_2_-glycoprotein1 IgG/IgM) were analyzed by multiplexed bead technology (Luminex) using BioPlex 2200 system (Bio-Rad, Hercules, CA, USA) according to the specifications of the manufacturer. Lupus anticoagulant (LA) was determined using a modified Dilute Russel Viper Venom method (Biopool, Umeå, Sweden) and Bioclot lupus anticoagulant. Complement factors C1q, C4, C3, and C3dg were all measured at Karolinska University Hospital. Complement factors C3a and the fluid-phase terminal complement complex, consisting of the components C5b, C6, C7, C8, and C9 and the S-protein were measured by sandwich ELISA as described earlier [13, 14], in addition C2 concentrations were measured by electroimmunoassay [15]. Rheumatoid factor (RF) IgM/IgA/IgG was analyzed by enzyme immune assay using Phadia 2500 (Elia, Phadia Thermo Fisher Scientific, Uppsala, Sweden). The detection range was 0.4–≥ 214 IU/ml for RF-IgM, 0.4–200 IU/ml for RF-IgA, and 0–600 μg/ml for RF-IgG.

### Protein profiling by affinity-based proteomics

#### Protein selection

**Fig. 1:**
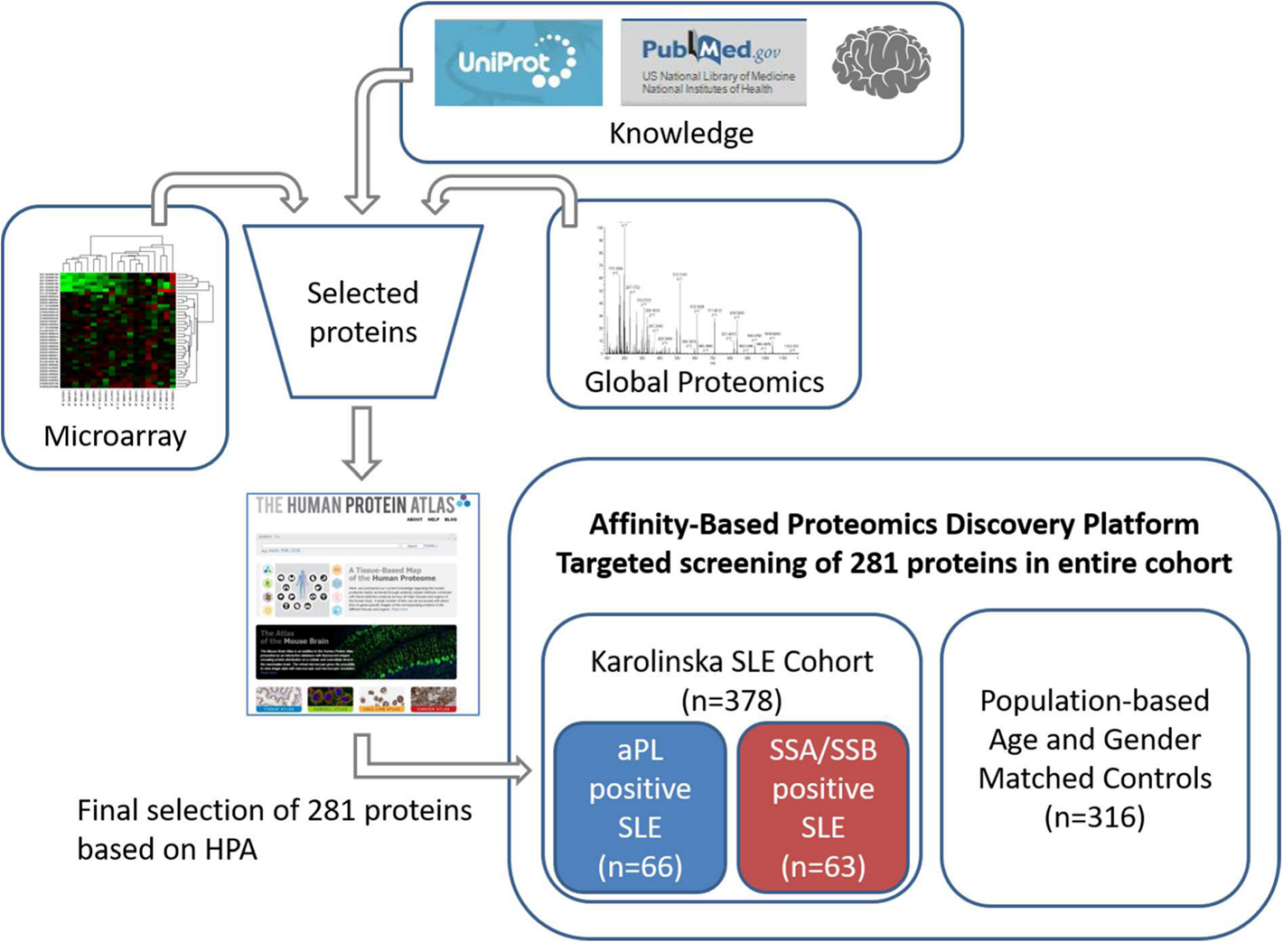
Analysis workflow. A list of protein targets was generated based on literature search, previous knowledge, genes known to be upregulated in SLE (microarray data) and on data from global LC-MS-based proteomics analysis on a selection of samples (*n* = 27). This list was searched through the Human Protein Atlas for available high-quality antibodies towards these proteins. Finally, a selection of antibodies towards 281 proteins was used for affinity proteomic analysis and screening of the Karolinska SLE cohort. The Karolinska SLE cohort comprised 378 SLE patients and 316 age- and gender-matched population-based controls. According to our autoantibody-based definition, the cohort consists of 66 aPL+ SLE and 63 SSA/SSB+ SLE patients

Selection of proteins (Fig. 1) was based on previous published results identifying candidate biomarkers in SLE, myositis and general inflammation. A list of proteins shown to be upregulated in SLE compared to controls based on microarray data [16] were included in the list of protein targets. In addition, our own global, untargeted MS-based proteomics analysis (Additional file 1) provided additional biomarker candidates. The study was subsequently performed against those proteins to which high-quality antibodies were available within the Human Protein Atlas (HPA) project [17]. The HPA contains the majority of all human protein-coding genes and polyclonal antibodies have been produced targeting protein fragments of the corresponding proteins. These fragments are selected to have as low sequence identity as possible to other human proteins. A number of 281 proteins were included, targeted by 367 antibodies, i.e., several proteins were detected by more than one antibody.

#### Suspension bead array technology

Protein profiles were generated for the selected 367 antibodies using a suspension bead array methodology, as previously described [18]. In brief, the 367 HPA antibodies were attached to color-coded magnetic beads, then incubated with 45 μl diluted and biotinylated EDTA-plasma, followed by an addition of streptavidin-conjugated R-phycoerythrin (Invitrogen), and finally analyzed using a FlexMap3D instrument (Luminex Corp.). A more detailed description can be found in Additional file 2.

### Data analysis

Univariate statistical testing, correlation analysis, and receiver operating characteristic (ROC) analysis were performed using R [19]. Wilcoxon rank-sum test were used to single out potential biomarkers in the study with Bonferroni correction for multiple testing. The number of missing data points for each variable is shown in Additional file 3. Variables with an absolute value of Spearman’s correlation coefficient (*r*_s_) between 0.20 and 0.39 were reported as weak correlations and 0.40 and 0.59 was interpreted as moderate correlations, *r*_s_ between 0.60 and 0.79 as strong correlations, and between 0.80 and 1.0 as very strong correlations.

## Results

### Clinical and serological characterization of SLE subgroups

Sixty-six patients belonged to the aPL+ and 63 to the SSA/SSB+ SLE subgroup (Fig. 1). Only three patients (0.03%) were positive for both LA and SSA/SSB and not assigned to any of the two subgroups. Secondary Sjögren’s syndrome (sSS), diagnosed according to the American-European Consensus criteria [20], was present in 34 patients (54%) in the SSA/SSB+ subgroup. In the aPL+ subgroup, 28 patients (42%) were found to have secondary APS (sAPS) according to the Miyakis definition of APS [4].

**Fig. 2:**
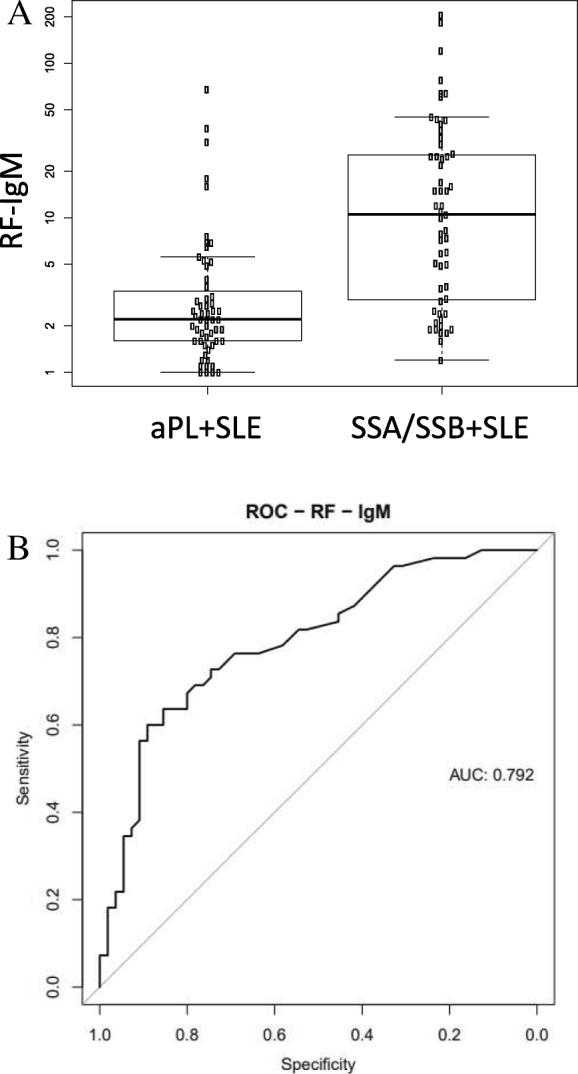
RF-IgM were found to be significantly increased in the SSA/SSB+ SLE subgroup compared to aPL+ subgroup (*p* = 1.3E-7). The levels of RF-IgM comparing the two subgroups are shown in (**a**), and the ROC curve are shown in (**b**) with an AUC of 0.79

Several clinical and serological measurements differed between the two suggested main subgroups (Table 1). Nephritis was less common in the SSA/SSB+ group (21% vs. 48%). Disease activity as measured by SLEDAI-2K was higher in the aPL+ group while SLAM scores did not differ. SLICC scores were also slightly higher in the aPL+ subgroup, despite similar disease duration in both groups. In the SSA/SSB+ group, we observed higher levels of total IgG but lower levels of total IgM than in the aPL+ group. RF was increased in the SSA/SSB+ subgroup and the levels of RF-IgM differentiated the SSA/SSB+ subgroup from the aPL+ subgroup with a ROC AUC of 0.79 (Fig. 2). The number of leucocytes was lower in the SSA/SSB+ subgroup, a difference partly due to the increase of neutrophils in the aPL+ SLE subgroup. Biomarkers associated with systemic inflammation, i.e., fibrinogen, α-1 antitrypsin, and triglycerides, were increased in aPL+ SLE. Complement factor 2 (C2), i.e., a component of the classical pathway of complement activation, was higher (*p* < 0.0001) in the SSA/SSB+ as compared to the aPL+ subgroup. A trend towards higher levels of complement factors C1q, C3, and C4 was also observed in the SSA/SSB+ subgroup. In addition, we noted that a degradation fragment of complement factor 3 (C3dg), a measure of complement activation [21], was higher in the aPL+ group (*p* < 0.0001) suggesting more complement activation in the aPL+ than in the SSA/SSB+ subgroup. More patients in the aPL+ subgroup were treated with warfarin (*n* = 21 vs. *n* = 5) and selective serotonin reuptake inhibitors (SSRI) (*n* = 7 vs. *n* = 4). However, 68% of the patients in the aPL+ SLE subgroup were not on warfarin, and with respect to other medications, there were no significant differences between the two suggested subgroups (Additional file 4).

**Table 1:** Clinical and serological characterization of the SLE patients, matched controls, and suggested aPL+ and SSA/SSB+ SLE subgroups

	Controls *n* = 316 ^1^	SLE *n* = 378 ^1, 2^	aPL+ SLE *n* = 66 ^1^	SSA/SSB+ SLE *n* = 63 ^1^	Control vs. aPL+ SLE *p* value ^3^	Control vs. SSA/SSB+ SLE *p* value ^3^	aPL+ vs. SSA/SSB+ SLE *p* value ^3^
Demographic/clinical							
Sampling age	48.1 (11.5)	47.7 (11.8)	47.9 (9.99)	48.6 (11.6)	8.7E−01	7.9E−01	9.8E−01
Gender (%female)	92.1 (−)	87.3 (−)	83.3 (−)	84.1 (−)	*3.6E−02*	5.7E−02	1.0E+00
Nephritis (%yes)	0.316 (−)	42.3 (−)	48.5 (−)	20.6 (−)	*5.0E−27*	*3.0E−10*	*1.5E−03*
SLEDAI-2K	–	4 (3.5)	4 (3)	3 (2.75)	–	*–*	*1.6E−02*
SLAM	–	6 (3)	6 (3.5)	7 (2.5)	–	–	7.4E−01
SLICC total	–	1 (1)	2 (1.5)	1 (1)	–	–	*1.1E−02*
Disease duration	–	11.6 (8.81)	10.3 (9.52)	8.42 (9.01)	–	–	8.2E−01
Serological/biochemical							
A1-antitrypsin (g/l)	1.4 (0.15)	1.4 (0.15)	1.5 (0.2)	1.4 (0.125)	*8.7E−06*	4.6E−01	*3.3E−03*
Apo A (g/l)	1.7 (0.25)	1.5 (0.2)	1.4 (0.15)	1.4 (0.15)	*3.3E−07*	*5.3E−06*	5.0E−01
Apo B (g/l)	0.81 (0.155)	0.81 (0.135)	0.83 (0.125)	0.79 (0.159)	1.5E−01	7.9E−01	2.2E−01
C1q (% of normal)	–	103 (17.6)	101 (19.2)	105 (22)	–	–	5.5E−01
C2 (% of normal)	–	118 (25.8)	105 (24)	144 (28.2)	–	–	*1.4E−05*
C3 (g/l)	1.04 (0.145)	0.87 (0.165)	0.8 (0.135)	0.91 (0.149)	*4.6E−14*	*5.5E−09*	1.8E−01
C3a (μg/l)	298 (1620)	270 (3586)	759 (1582)	1102 (1852)	6.8E−01	4.3E−01	3.6E−01
C3dg (mg/l)	–	7.6 (2.05)	9.2 (1.93)	6.1 (1.58)	–	*–*	*2.7E−05*
C4 (g/l)	0.21 (0.04)	0.15 (0.05)	0.125 (0.045)	0.15 (0.0338)	*1.0E−17*	*3.9E−13*	9.5E−02
TCC (AU/l)	30 (25)	59 (153)	65 (57)	78 (74)	*2.7E−04*	*3.4E−05*	2.7E−01
Creatinine (μmol/l)	66 (11)	69 (80)	72 (83)	69 (70)	*5.6E−06*	*1.1E−02*	8.2E−02
SLEDAI-2K Proteinurea = 4 (*n*)	–	39 (10%)	11 (17%)	3 (5%)	–	–	*3.1E−02*
Fibrinogen (g/l)	3.8 (0.6)	4.1 (0.8)	4.5 (0.65)	4.1 (0.8)	*4.4E−08*	9.9E−02	*8.8E−03*
Homocysteine (μmol/l)	9.3 (1.48)	12 (2.69)	13.6 (3.2)	11.3 (2.32)	*3.6E−13*	*1.9E−08*	1.1E−01
Hs CRP (mg/l)	0.96 (0.825)	1.7 (2.22)	2.5 (3.73)	1.8 (2.26)	*1.5E−08*	*1.7E−04*	5.3E−02
ESR	8.5 (8)	19 (22)	31 (25)	29 (30)	*2.2E−16*	*2.2E−16*	7.5E−1
IgA (g/l)	2.1 (0.674)	2.8 (0.938)	2.65 (1.04)	2.9 (1.2)	*3.8E−03*	*1.6E−04*	4.8E−01
IgG (g/l)	10.9 (1.35)	12.8 (3.18)	12.6 (3.19)	16 (5.18)	*1.0E-05*	*6.1E−12*	*5.4E−03*
IgM (g/l)	1 (0.393)	0.93 (0.414)	1.2 (0.526)	0.82 (0.268)	7.1E−01	*1.0E−04*	*8.1E−03*
RF-IgA (IU/ml)	3.7 (1.8)	5.3 (3.8)	5.7 (2.4)	29.5 (30.6)	*3.1E−03*	*1.3E−10*	*1.5E−05*
RF-IgG (μg/ml)	7.5 (3.2)	11.0 (6.5)	11.0 (6.4)	26.0 (20.5)	*2.3E−03*	*6.4E−10*	*9.1E−04*
RF-IgM (IU/ml)	1.1 (0.6)	1.3 (2.2)	1.2 (0.9)	10.5 (14.6)	0.7	*4.2E−15*	*2.1 E−08*
Leucocytes (× E9/l)	5.5 (1)	5.1 (1.55)	5.5 (1.7)	4.8 (1.35)	6.4E−01	*4.9E−05*	*1.8E−02*
Lymphocytes (× E9/l)	1.8 (0.3)	1.1 (0.35)	1.1 (0.362)	0.9 (0.275)	*9.6E−17*	*5.7E−23*	1.5E−01
Neutrophils (× E9/l)	3 (0.8)	3.2 (1.3)	3.55 (1.46)	3.2 (1.17)	*3.4E−02*	2.8E−01	*2.7E−02*
Platelets (× E9/l)	260 (36.5)	234 (50)	219 (47)	225 (61.8)	*7.4E−06*	*8.0E−03*	1.9E−01
TG mmol/l)	0.77 (0.275)	1 (0.355)	1.4 (0.385)	0.9 (0.34)	*1.2E−10*	*1.1E−02*	*1.5E−03*
VCAM (μg/l)	364 (71)	388 (95)	444 (93.9)	367 (91.4)	*2.1E−04*	4.4E−01	5.7E−02

^1^Data reported as median and IQR

^2^This column represents the data from the entire SLE cohort including the aPL+ and SSA/SSB+ SLE subgroups

^3^*p* value obtained by Wilcoxon rank-sum test or Fisher’s exact test. *p* values < 0.05 are highlighted in italics

### Protein profiling of SLE subgroups by affinity-based proteomics

**Fig. 3:**
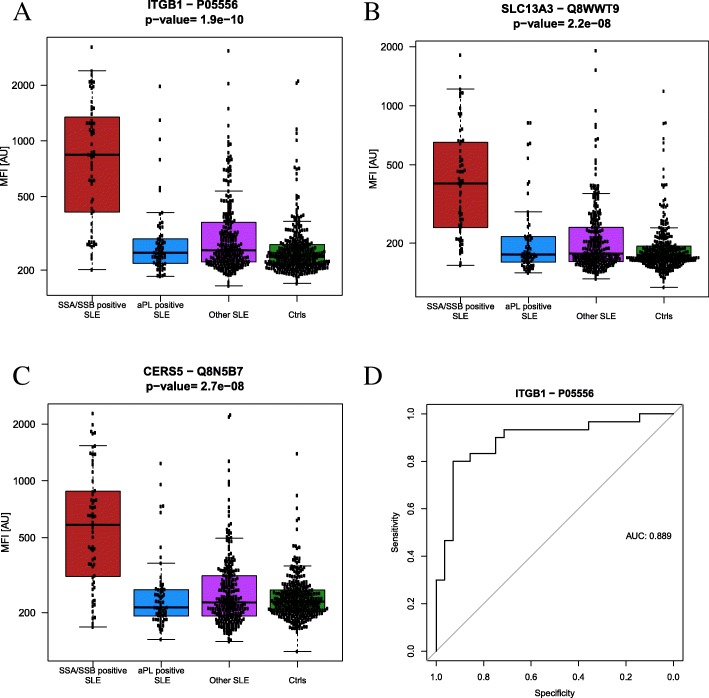
ITGB1 was the protein with the highest prediction power for separating aPL+ SLE from the SSA/SSB+ SLE subgroup. The protein levels of ITGB1 in relation to other SLE patients and controls are shown. Proteins with the lowest *p* values comparing aPL+ and SSA/SSB+ SLE subgroup were **a** ITGB1 (*p* = 1.9e−10), **b** SLC13A3 (*p* = 2.2e−8), and **c** CERS5 (*p* = 2.7e−8). A ROC curve for ITGB1 (**d**) obtained an AUC of 0.89

The protein profiles obtained by affinity proteomics for the two SLE subgroups revealed significant differences in several proteins (Table 2). Integrin beta-1 (ITGB1, Fig. 3a), solute carrier family 13 member 3 (SLC13A3, Fig. 3b), and ceramide synthase 5 (CERS5, Fig. 3c) were the proteins with the lowest *p* values, and all three were elevated in the SSA/SSB+ subgroup. The best predictor for distinguishing between aPL+ SLE and SSA/SSB+ SLE in ROC analysis was ITGB1 (Fig. 3a), showing an AUC of 0.89 (Fig. 3d). The proteins reported in Table 2 did not correlate (*r*_s_ < 0.2) with measures of disease activity.

**Table 2:** Affinity-based proteomics results comparing aPL+ SLE (*n* = 66) vs SSA/SSB+ SLE (*n* = 63) subgroup. The proteins with the lowest *p* values comparing the two suggested subgroups are shown

Gene	Uniprot ID	Protein name	Protein function^1^	*p* value ^2^aPL+ vs SSA/SSB+ SLE	Fold change^3^ SSA/SSB+ vs. aPL+ SLE
ITGB1	P05556	Integrin beta-1	Cell adhesion, host-virus interaction, integrin	1.90E*−*10	*3.4*
SLC13A3	Q8WWT9	Solute carrier family 13 member 3	Ion transport, sodium transport	2.20E*−*08	*2.3*
CERS5	Q8N5B7	Ceramide synthase 5	Lipid biosynthesis/metabolism, sphingolipid metabolism	2.70E*−*08	*2.7*
MSX2	P35548	Homeobox protein MSX-2	Osteogenesis, transcription, transcription regulation	2.80E*−*08	1.5
F3	P13726	Tissue factor. Coagulation factor III	Blood coagulation, hemostasis	3.60E*−*08	1.7
HSP90AA1	P07900	Heat shock protein HSP 90-alpha	Stress response, chaperon	5.00E*−*08	*2.6*
MMP8	P22894	Neutrophil collagenase. Metalloproteinase 8	Collagen degradation	5.40E*−*08	*2.1*
CTSB	P07858	Cathepsin B	Thiol protease	6.10E*−*08	1.3
MMP10	P09238	Stromelysin-2	Collagen degradation	1.30E*−*07	1.9
YARS	P54577	Tyrosine--tRNA ligase	Protein biosynthesis, aminoacyl-tRNA synthetase	5.30E*−*07	*2.1*
SELE	P16581	E-selectin	Cell adhesion	7.70E*−*07	*2.4*
FMO1	Q01740	Dimethylaniline monooxygenase [N-oxide-forming] 1	Catalyzes the *N*-oxygenation of secondary and tertiary amines	9.90E*−*07	1.8
SAMD8	Q96LT4	Sphingomyelin synthase-related protein 1	Lipid metabolism, sphingolipid metabolism	3.40E*−*06	*2.1*
ETNPPL	Q8TBG4	Ethanolamine-phosphate phospho-lyase	Aminotransferase, lyase, transferase	5.70E*−*06	1.3
ARID2	Q68CP9	AT-rich interactive domain-containing protein 2. BAF200	Transcription, transcription regulation, chromatin regulator	8.90E*−*06	1.9
ETV7	Q9Y603	Transcription factor ETV7	Repressor	1.20E*−*05	1.4
CD40	P25942	Tumor necrosis factor receptor superfamily member 5. B-cell surface antigen CD40	Receptor for TNFSF5/CD40LG	1.50E*−*05	1.6
KRT7	P08729	Keratin. type II cytoskeletal 7	Viral process, blocks interferon-dependent interphase and stimulates DNA synthesis in cells	4.10E*−*05	0.8
TYK2	P29597	Non-receptor tyrosine-protein kinase TYK2	Kinase, transferase, tyrosine-protein kinase	6.70E*−*05	1.4
REN	P00797	Renin. Angiotensinogenase	Aspartyl protease, hydrolase, protease	7.30E*−*05	1.4
APCS	P02743	Serum amyloid P-component	DNA-binding and opsonizing protein	9.10E*−*05	0.9
CYR61	O00622	Insulin-like growth factor-binding protein 10	Growth factor binding, heparin-binding	0.00013	0.9
CLDN16	Q9Y5I7	Claudin 16	Ion transport, transport	0.00014	1.7
GOT1	P17174	Aspartate aminotransferase	Aminotransferase, transferase	0.00017	1.3
EGF	P01133	Pro-epidermal growth factor	Growth factor	0.00027	1.2

^1^Protein function/biological process according to www.uniprot.org [54]

^2^*p* value obtained by Wilcoxon rank-sum test and Bonferroni correction for multiple testing

^3^Fold change is reported comparing the two subgroups. Values above 1 means upregulated in the SSA/SSB+ subgroup and values below 1 that the protein was downregulated in the SSA/SSB+ subgroup. Mean fold change of more than 100% is highlighted in italics

Among the 25 proteins with the lowest *p* values comparing the two subgroups (Table 2), three proteins were downregulated in the SSA/SSB+ subgroup, i.e., keratin, type II cytoskeletal 7 (KRT7), serum amyloid P-component (APCS or SAP), and protein CYR61 (CYR61). Seven of the 25 proteins showed weak positive or negative correlation to RF-IgM, ten correlated strongly (*r*_s_ > 0.60), but none of the proteins showed very strong correlation to RF-IgM. The correlation between RF-IgM and all proteins analyzed (*n* = 281) is shown in Additional file 5, and *r*_s_ for the 25 proteins is shown in Additional file 6. When studying the correlation between RF-IgM and the 25 proteins in data obtained for the controls, excluding data from SLE patients, five of these proteins were found not to correlate (*r*_s_ < 0.2) to RF-IgM, i.e., keratin, type II cytoskeletal 7 (KRT7 or sarcolectin), glutamic-oxaloacetic transaminase 1 (GOT1 or aspartate aminotransferase, cysteine aminotransferase), ethanolamine phosphate phospholyase (ETNPPL or alanine--glyoxylate aminotransferase 2-like 1, AGXT2L1), renin (REN), and dimethylaniline monooxygenase 1 (FMO1).

## Discussion

To shed light on the heterogeneity of SLE we built on previous observations [8, 9], we designed a simple and clinically useful subgroup definition based on autoantibody profiles. The SSA/SSB+ group was characterized by elevated levels of IgG and depressed levels of IgM, in line with a recent report from our group [22], and lower frequency of nephritis (21% vs. 48%). Others and we have previously noted that SSA/SSB antibodies are associated with a less severe SLE, with less renal manifestations and reduced risk for cardiovascular disease and mortality [7, 23, 24]. It is known that SSA/SSB autoantibodies in patients with SS are associated with RF [25] and we could confirm higher levels of RF of IgG, IgM, and IgA isotype in the SSA/SSB+ group.

Signs of systemic inflammation were found to be increased in the aPL+ subgroup, which is in agreement with previous studies of primary and secondary APS [26, 27]. In the aPL+ group, indicators of complement activation (lower C2 and higher C3dg) were pronounced. These observations are in line with several previous reports. Oku et al. reported that hypocomplementemia is common in primary APS [28] and C3 and C4 levels have been reported to be decreased in APS [29]. Furthermore, complement inhibition prevented aPL-induced pregnancy loss and thrombosis in mice [30], and eculizumab, an antibody that binds to complement factor 5 and blocks terminal complement activation, reversed catastrophic APS [31]. Together these observations indicate that complement activation is a feature in APS as well as in the aPL+ SLE. Therefore, complement inhibition may be a more targeted approach of treatment for this subgroup of SLE patients.

A large effort was put in the selection of proteins, which is crucial to obtain informative protein profiles by affinity-based proteomics [18]. When applying affinity-based proteomics, 25 proteins could distinguish between the subgroups (*p* < 0.001). The protein with the best separation power (lowest *p* value) was integrin beta-1 (ITGB1), followed by solute carrier family 13 member 3 (SLC13A3) and ceramide synthase 5 (CERS5), and all three were elevated in the SSA/SSB+ group. ITGB1 (or CD29) is a protein that enhances autoreactive T cell activation and has been shown to be elevated in SLE patients with active disease [32]. SLC13A3 is an ion transporting plasma membrane protein enriched in kidney. It has been reported as a genomic biomarker in mice with a progressive loss of kidney function, and the protein expression was increased in human biopsies from patients with severe chronic kidney disease, stage III/IV [33]. The role of SLC13A3 in plasma vs tissue and its role in SLE merit further investigation. CERS5 is an enzyme involved in the sphingolipid metabolism. It catalyzes the formation of dihydroceramide and is known to suppress phosphatidylcholine biosynthesis [34]. Ceramides are signaling molecules affecting the immune system [35] and are involved in endothelial dysfunction [36]. In CERS5 knock-out mice, the cellular C16:0 sphingolipid pool is decreased [37], and in our previous work, we showed that C16:0 ceramide was elevated in SLE compared to controls, normalized after immunosuppressive treatment, and that it was associated with higher disease activity [38].

Most of the proteins differentiating the two groups were increased in SSA/SSB+ SLE, and only three of these proteins were decreased compared to the aPL+ subgroup, i.e., serum amyloid P-component (APCS or SAP), insulin-like growth factor-binding protein 10 (CYR61), and keratin, type II cytoskeletal 7 (KRT7 or sarcolectin). APCS is an acute phase protein structurally related to C-reactive protein. It is involved in clearance of dead cells [39] and might be associated with atherothrombosis [40], a known feature associated with aPL in SLE [10]. CYR61 is known to be increased in an inflammatory state of SLE [41], which is in line with our observation of increased systemic inflammation in aPL+ SLE patients. The third protein with decreased levels in SSA/SSB+ subgroup was KRT7, a protein that blocks interferon signaling [42].

Primary SS is known to be associated with chronic type 1 IFN response [43–45]. The association between aPL and IFN signature is more debated, and there are just a few studies reporting an IFN signature in primary APS [46]. We recently demonstrated that aPL+ SLE patients had lower levels of circulating INF-α as compared to other lupus patients [47]. As mentioned, we detected lower levels of KRT7 in SSA/SSB+ SLE suggesting increased IFN signaling in this subgroup. In addition, we detected elevated levels of non-receptor tyrosine-protein kinase TYK2 (TYK2). TYK2 is known to initiate type I IFN signaling and is associated with the IFN-α receptor 1 [48]. *TYK2* has also been reported as a susceptibility gene in SLE [49]. Increased levels of transcription factor ETV7 (ETV7, or ETS translocation variant 7, or TEL2) in SSA/SSB+ SLE further support a more pronounced IFN signature on the protein level in this subgroup compared to aPL+ subgroup. *ETV7* is an IFN-α-stimulated gene [50], also known to induce IFN-γ [51]. Furthermore, AT-rich interactive domain-containing protein 2 (ARID2 or BAF200), a subunit of the polybromo-associated barrier-to-autointegration factor (PBAF) chromatin-remodeling complex, which is known to regulate the expression of multiple interferon-responsive genes [52], was elevated in the SSA/SSB+ SLE subgroup. These findings support increased IFN signaling in the SSA/SSB+ subgroup and suggest that IFN-blocking therapy might be favorable in this subgroup.

One limitation with our study, and a natural obstacle in many studies when comparing different groups of patients, is the difference in treatment between groups. For ethical reasons, it was not possible to withdraw treatment to perform this study. As expected, more patients were on warfarin in the aPL+ subgroup (31% vs. 8%). However, most of the aPL+ patients did not receive this medication.

Another limitation might be secondary binding problems that are present in all immunoassays but is usually neglected and seldom discussed. RF could be an interfering factor in the affinity proteomics, and since it is more pronounced in the SSA/SSB+ subgroup, this might influence the results. RF may enhance the signal in the biotinylated sample if RF binds to the targeted protein, to the assay antibody directly, to the IgG in plasma that interact with the targeted protein, or to the assay antibody on the bead. However, this problem is rather unlikely since RF is an antibody binding to the Fc region of IgG [53] and we have applied sample dilution with appropriate buffers to limit secondary binding. For the majority of the proteins in Table 2, we observed a weak or moderate correlation to RF-IgM and three of these even showed negative correlations, i.e., RF-IgM did not enhance the signal for these proteins. We believe that the differences in protein profiles between the two subgroups reflect actual differences and that the correlation to RF-IgM is a biological phenomenon and not represent methodological artifacts.

## Conclusion

We suggest that the present SLE diagnosis harbors at least two main subgroups, here defined by autoantibody profile as aPL+ and SSA/SSB+. Several new candidate biomarkers were identified in this work highlighting differences in molecular signature between the two subgroups implying possible differences in pathogenesis and treatment perspectives. We suggest that IFN-directed therapy is more likely to be efficient in the SSA/SSB+ subgroup since this subgroup seems to have an activated interferon system. Complement activation and systemic inflammation were more common in the aPL+ group suggesting that therapy targeting complement is more effective in this subgroup. It is important to identify subgroups with increased risk of long-term co-morbidities, e.g., the increased risk of vascular events, and if such patients can be identified at an early stage and treated prophylactically, severe outcomes may be prevented.

## Additional files

Additional file 1: A more detailed description of the MS proteomic method is described. (PDF 289 kb)

Additional file 2: A more detailed description of the affinity-based proteomic method is described. (PDF 370 kb)

Additional file 3: **Table S1.** The number of missing data points for each variable is shown. (PDF 668 kb)

Additional file 4: **Table S2.** Medications for the patients are reported. (PDF 526 kb)

Additional file 5: **Figure S1.** The correlation between RF-IgM and all proteins analyzed. (PDF 284 kb)

Additional file 6: **Table S3.** The correlation between RF-IgM and the 25 proteins in Table 2 are shown. (PDF 256 kb)

## Abbreviations

Anti-dsDNA: Anti-double stranded DNA
Anti-La: Anti-Sjögren’s-syndrome-related antigen B
Anti-Ro: Anti-Sjögren’s-syndrome-related antigen A
APCS: Serum amyloid P-component
aPL: Anti-phospholipid
APS: Anti-phospholipid syndrome
ARID2: AT-rich interactive domain-containing protein 2 or BAF200
C1q: Component 1q (in complement system)
C2: Component 2 (in complement system)
C3: Component 3 (in complement system)
C3dg: Component 3 degradation product (in complement system)
C4: Component 4 (in complement system)
CD40: Tumor necrosis factor receptor superfamily member 5
CERS5: Ceramide synthase 5
CLDN16: Claudin 16
CTSB: Cathepsin B
CYR61: Insulin-like growth factor-binding protein 10
EDTA: Ethylenediaminetetraacetic acid
EGF: Pro-epidermal growth factor
ELISA: Enzyme-linked immunosorbent assay
ETNPPL: Ethanolamine-phosphate phospholysate
ETV7: Transcription factor ETV7
F3: Tissue factor. Coagulation factor III
FMO1: Dimethylaniline monooxygenase
GOT1: Glutamic-oxaloacetic transaminase 1
HPA: Human Protein Atlas
Hs CRP: High-sensitivity C-reactive protein
HSP90AA1: Heat shock protein HSP 90α
IgG: Immunoglobulin G
IgM: Immunoglobulin M
ITGB1: Integrin beta-1
KRT7: Keratin, type II cytoskeletal 7
LA: Lupus anticoagulant
LC-MS/MS: Liquid chromatography tandem mass spectrometry
m/z: Mass to charge ratio
MMP10: Stromelysin-2
MMP8: Metalloproteinase 8
MSX2: Homeobox protein MSX-2
PBAF: Polybromo-associated barrier-to-autointegration factor
REN: Renin
SAMD8: Sphingomyelin synthase-related protein 1
SELE: E-selectin
SLAM: Systemic Lupus Activity Measurement
SLC13A3: Solute carrier family 13 member 3
SLE: Systemic lupus erythematosus
SLEDAI-2K: Systemic Lupus Erythematosus Disease Activity Index 2000
SLICC: Systemic Lupus International Collaborating Clinics
Sm/RNP: Anti-Smith/ribonuclear
SS: Sjögren’s syndrome
SSA/SSB: Sjögren’s syndrome antigen A/B
TG: Triglycerides
TYK2: Non-receptor tyrosine-protein kinase TYK2
YARS: Tyrosine--tRNA ligase
