# Supplementary material for: Two subgroups in systemic lupus erythematosus with features of antiphospholipid or Sjögren’s syndrome differ in molecular signatures and treatment perspectives
Source: Arthritis Res Ther. 2019 Feb 18;21:62. doi: 10.1186/s13075-019-1836-8 (PMC6378708; doi:10.1186/s13075-019-1836-8)
Supplement: Supplementary file 1 — A more detailed description of the MS proteomic method is described. (PDF 289 kb) [file 13075_2019_1836_MOESM1_ESM.pdf]

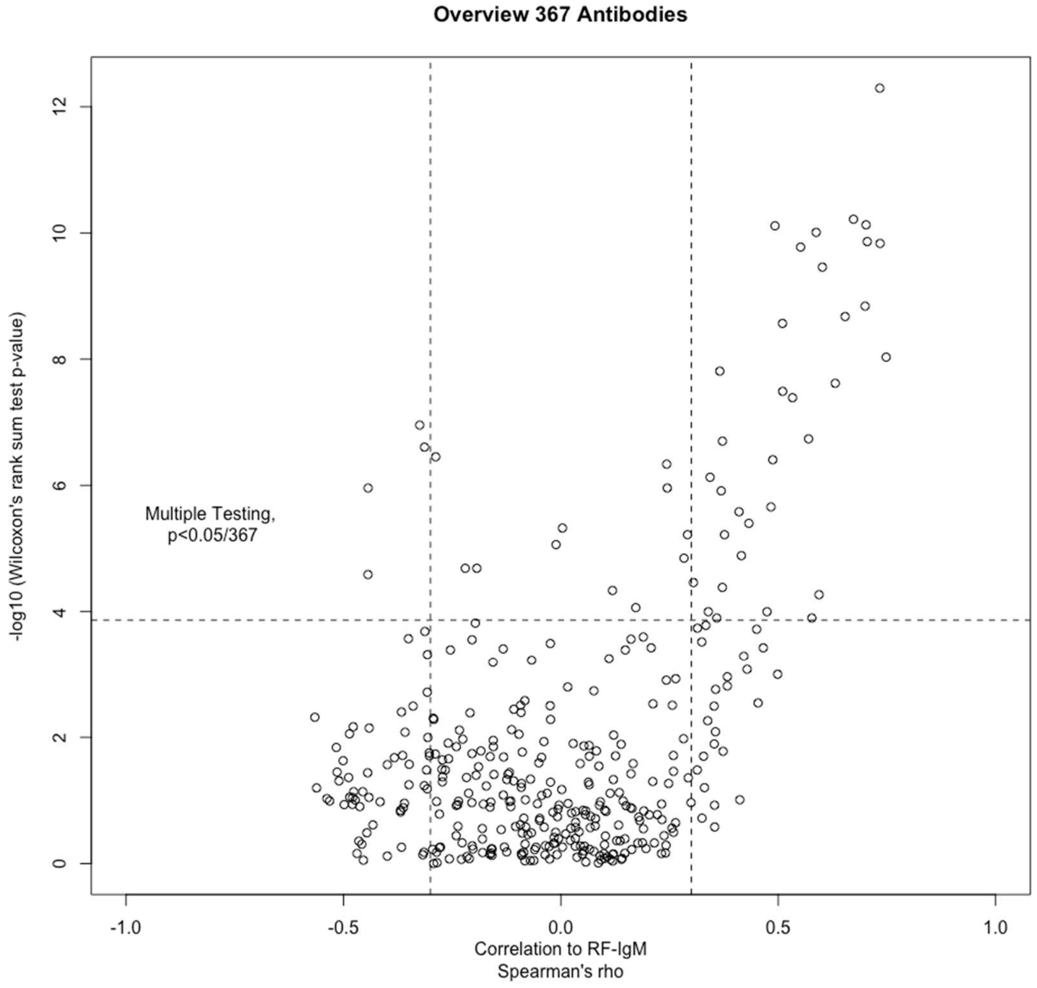

Supplementary Figure S-1. P-values obtained when comparing aPL+ and SSA/SSB+ subgroups for all 367 antibodies in the assay are plotted as a function of their correlation (Spearman's rho) to RF-IgM. Several proteins with low p-values are shown to correlate to RF-IgM (positive correlation in upper right and negative in upper left corner) and there are proteins that do not ($r_s < |0.2|$). Correlations between 0.40 and 0.59 are interpreted as moderate correlations. Proteins above the horizontal cut-off line (Bonferroni corrected $p<0.05$) are reported in **Table 2**.
