# Supplementary material for: Two subgroups in systemic lupus erythematosus with features of antiphospholipid or Sjögren’s syndrome differ in molecular signatures and treatment perspectives
Source: Arthritis Res Ther. 2019 Feb 18;21:62. doi: 10.1186/s13075-019-1836-8 (PMC6378708; doi:10.1186/s13075-019-1836-8)
Supplement: Supplementary file 2 — A more detailed description of the affinity-based proteomic method is described. (PDF 370 kb) [file 13075_2019_1836_MOESM2_ESM.pdf]

Supplementary Table S-1. Showing number of subjects with missing values for each clinical and serological measurement for the controls, SLE patients and the two suggested subgroups of aPL+ SLE and SSA/SSB+ SLE.

**Missing Values**	**Controls n=316**	**SLE n=378**	**aPL+ SLE n=66**	**SSA/SSB+ SLE n=63**
**Sampling Age**	0	0	0	0
**Gender**	0	0	0	0
**Nephritis**	3	0	0	0
**SLEDAI-2K**	316	0	0	0
**SLAM**	316	0	0	0
**SLICC.total**	316	0	0	0
**Disease Duration**	316	0	0	0
**Acetylsalicylic Acid**	313	376	66	63
**Azathioprin**	316	327	55	58
**Calcium and D supplementation**	313	343	60	58
**Cyclophosphamide**	316	377	66	63
**D vitamin**	2	9	0	2
**Esomeprazol**	314	368	64	62
**Folic Acid**	315	354	60	61
**Furosemide**	315	352	62	62
**Hyrdoxychloroquine**	316	311	57	51
**Ibuprofen**	312	365	62	60
**Karbamazepin**	316	374	66	62
**Levothyroxine**	296	335	60	55
**Methotrexate**	316	367	64	63
**Mycophenolate**	316	351	63	60
**Paracetamol**	304	319	59	54
**Rituximab**	314	69	11	8
**SNRI**	0	0	0	0
**SSRI**	0	0	0	0
**Warfarin**	315	339	45	58
**A1-antitrypsin**	4	7	3	0
**Apo A**	13	76	13	9
**Apo B**	13	76	13	9
**C1q**	316	22	2	2
**C2**	316	43	9	5
**C3**	0	9	2	1
**C3dg**	316	121	24	17
**C4**	0	9	2	1
**TCC**	0	0	0	0
**Creatinine**	0	0	0	0
SLEDAI-2K Proteinurea =4	316	0	0	0
**ESR**	8	15	3	3
**Fibrinogen**	8	11	3	0
**Homocystein**	13	76	13	9
**Hs CRP**	11	2	0	0
**IgA**	4	0	0	0
IgG	4	0	0	0
IgM	4	0	0	0
Rf-IgM	95	29	8	5

Rf-IgA	97	53	7	19
Rf-IgG	112	53	8	10
Leucocytes	0	1	1	0
**Lymphocytes**	0	3	2	0
**Neutrophils**	0	3	2	0
**Platelets**	0	1	1	0
**TG**	0	1	0	0
**VCAM**	6	76	12	10
