# Supplementary material for: Two subgroups in systemic lupus erythematosus with features of antiphospholipid or Sjögren’s syndrome differ in molecular signatures and treatment perspectives
Source: Arthritis Res Ther. 2019 Feb 18;21:62. doi: 10.1186/s13075-019-1836-8 (PMC6378708; doi:10.1186/s13075-019-1836-8)
Supplement: Supplementary file 3 — Table S1. The number of missing data points for each variable is shown. (PDF 668 kb) [file 13075_2019_1836_MOESM3_ESM.pdf]

**Supplementary Table S-2**. Number of subject with reported medication among the SLE patients, matched controls, and the aPL+ and SSA/SSB+ SLE subgroups. Bonferroni corrected p-values < 0.05 are highlighted in bold.

| | Controls n=316 Median (IQR) [1] | SLE n=378 Median (IQR) [1] | aPL+ SLE n=66 Median (IQR) [1] | SSA/SSB+ SLE n=63 Median (IQR) [1] | Ctrls vs. aPL+ SLE p-value[2] | Ctrls vs. SSA/SSB+ SLE p-value[2] | aPL+ vs. SSA/SSB+ SLE p-value[2] |
|---|---|---|---|---|---|---|---|
| **Medication** | | | | | | | |
| Acetylsalicylic Acid | 3 | 2 | 0 | 0 | - | - | - |
| Azathioprin | 0 | 51 | 11 | 5 | **8.9E-09** | **1.5E-04** | 1.2E-01 |
| Calcichew | 3 | 35 | 6 | 5 | **1.8E-03** | **5.5E-03** | 7.6E-01 |
| Cyclophosphamide | 0 | 1 | 0 | 0 | - | - | - |
| D vitamin | 15 | 198 | 37 | 29 | **1.6E-19** | **3.7E-16** | 4.8E-01 |
| Esomeprazol | 2 | 10 | 2 | 1 | 1.5E-01 | 4.3E-01 | 1.0E+00 |
| Folic Acid | 1 | 24 | 6 | 2 | **2.0E-04** | 7.7E-02 | 2.7E-01 |
| Furosemide | 1 | 26 | 4 | 1 | **4.3E-03** | 3.1E-01 | 3.7E-01 |
| Hyrdoxychloroquine | 0 | 67 | 9 | 12 | **2.3E-07** | **1.2E-09** | 8.1E-01 |
| Ibuprofen | 4 | 13 | 4 | 3 | **3.9E-02** | 1.0E-01 | 1.0E+00 |
| Karbamazepin | 0 | 4 | 0 | 1 | - | 1.7E-01 | 4.9E-01 |
| Levothyroxine | 20 | 43 | 6 | 8 | 6.0E-01 | 2.0E-01 | 7.8E-01 |
| Methotrexate | 0 | 11 | 2 | 0 | **3.1E-02** | 1.0E+00 | 5.0E-01 |
| Mycophenolate | 0 | 27 | 3 | 3 | **5.6E-03** | **5.0E-03** | 1.0E+00 |
| Paracetamol | 12 | 59 | 7 | 9 | 6.3E-02 | **7.1E-03** | 8.0E-01 |
| Rituximab | 0 | 26 | 7 | 4 | **4.8E-09** | **3.7E-06** | 2.1E-01 |
| SNRI | 3 | 5 | 1 | 1 | **2.5E-02** | **2.5E-02** | 5.8E-02 |
| SSRI | 12 | 27 | 7 | 4 | **1.7E-06** | **2.5E-04** | **1.4E-02** |
| Warfarin | 1 | 39 | 21 | 5 | **2.4E-14** | **8.0E-04** | **3.8E-04** |
