# Supplementary material for: Two subgroups in systemic lupus erythematosus with features of antiphospholipid or Sjögren’s syndrome differ in molecular signatures and treatment perspectives
Source: Arthritis Res Ther. 2019 Feb 18;21:62. doi: 10.1186/s13075-019-1836-8 (PMC6378708; doi:10.1186/s13075-019-1836-8)
Supplement: Supplementary file 4 — Table S2. Medications for the patients are reported. (PDF 526 kb) [file 13075_2019_1836_MOESM4_ESM.pdf]

Supplementary Table S-3 Spearman's correlation between proteins in Table 2 (*i.e.*, proteins with the lowest p-values comparing the two subgroups) and RF-IgM are shown. Weak correlations are highlighted in bold.

**Gene**	**Uniprot ID**	**Spearman's correlation (r_s) RF-IgM vs. protein**
ITGB1	P05556	0.73
SLC13A3	Q8WWT9	0.67
CERS5	Q8N5B7	0.70
MSX2	P35548	0.49
F3	P13726	0.59
HSP90AA1	P07900	0.70
MMP8	P22894	0.73
CTSB	P07858	0.55
MMP10	P09238	0.60
YARS	P54577	0.70
SELE	P16581	0.65
FMO1	Q01740	0.51
SAMD8	Q96LT4	0.75
**ETNPPL**	Q8TBG4	**0.37**
ARID2	Q68CP9	0.63
ETV7	Q9Y603	0.51
CD40	P25942	0.53
**KRT7**	P08729	**-0.32**
TYK2	P29597	0.57
**REN**	P00797	**0.37**
**APCS**	P02743	**-0.31**
**CYR61**	O00622	**-0.29**
CLDN16	Q9Y5I7	0.49
**GOT1**	P17174	**0.24**
**EGF**	P01133	**0.34**
