# Supplementary material for: Two subgroups in systemic lupus erythematosus with features of antiphospholipid or Sjögren’s syndrome differ in molecular signatures and treatment perspectives
Source: Arthritis Res Ther. 2019 Feb 18;21:62. doi: 10.1186/s13075-019-1836-8 (PMC6378708; doi:10.1186/s13075-019-1836-8)
Supplement: Supplementary file 5 — Figure S1. The correlation between RF-IgM and all proteins analyzed. (PDF 284 kb) [file 13075_2019_1836_MOESM5_ESM.pdf]

Detailed Method Description: Mass Spectrometry-based Proteomics

Depletion of high abundance proteins

An aliquot of 50µl plasma were diluted 1:5 and centrifuged (10000g. 3min). 150µl were injected on to an Agilent Multiple Affinity Removal Column 4.6 × 100-mm (Agilent Technologies. www.agilent.se) according to the instructions of the manufacturer in order to deplete 6 high abundant proteins (albumin. IgG. IgA. antitrypsin. haptoglobin and transferrin). Protein containing fractions were concentrated using Millipore spin filter 10kDa. 5000 g. 20 minutes.

Digestion and 8-plex iTRAQ labeling

Protein content was measured using NanoDrop (Thermo Scientific. Wilmington. DE. USA). 20 µg of protein from each sample were reduced using DTT (final concentration 5 mM. at 56°C for 30 min) followed by alkylation by iodoacetamide (final concentration 0.015 M. for 1 hour at room temperature in darkness). Proteins were digested overnight at 37 °C using trypsin (modified sequencing grade Promega. Madison. WI) 1:20 enzyme:substrate. After digestion, peptides were labeled with 8-plex iTRAQ (Applied Biosystems. Foster City. CA. USA) according to the manufacturer's instructions. Excess of reagent were removed from the pooled sample using the SCX-cartridge (Strata SCX. Phenomenex. Torrence. CA. USA). The eluate was dried in a speed-vac.

Plasma proteomics using peptide pre-fractionation by ultra-narrow isoelectric focusing

Isoelectric focusing (IEF) involves the separation of proteins or peptides based on their isoelectric point, pI. In our workflow for mass spectrometry based quantification of complex proteomes we use high-resolution or narrow range isoelectric focusing of peptides prior to MS

analysis to reduce sample complexity and thereby increase proteome coverage [55, 56]. The method is compatible with iTRAQ labelling [57]. For the depleted plasma samples in this study we used an ultra-narrow pI range 4.20-4.45, an interval selected based on the theoretical pI distribution of tryptic peptides from the complement system proteins. Hence. protein identification and quantification was based on peptides within this pI interval.

Freeze dried peptide samples (estimation 140µg per 8-plex iTRAQ sample pool corresponding to 17.5 µg per individual sample) were dissolved in 160 µL rehydration solution containing 8 M urea and allowed to adsorb to the gel bridge by swelling overnight. Acidic (pI 4.20-4.45) ultra-narrow range 24 cm linear gradient IPG strips (GE Healthcare) were incubated overnight in 8 M rehydration solution containing 1% IPG pharmalyte pH 2.5–5.0 (GE Healthcare). Samples were applied to the IPG strips by the gel bridge (pH 3.7) at the cathode end and run as described [55]. After focusing the peptides were passively eluted into 72 contiguous fractions with MilliQ water using an in-house constructed IPG extractor robotics (GE Healthcare Bio-Sciences AB. prototype instrument). The resulting fractions were freeze dried and kept at -20 °C.

LC-ESI-LTQ-Orbitrap analysis – ultra narrow IPG plasma proteomics

Online LC-MS was performed using a hybrid LTQ-Orbitrap Velos mass spectrometer (Thermo Scientific). For each LC-MS/MS run the auto sampler (HPLC 1200 system Agilent Technologies) dispensed 8 µl of solvent A to the well in the 96 V plate mixed for 10 min. and proceeded to inject 3 µl. Samples were trapped on a C18 guard desalting column (Agilent) and separated on a 15-cm long C18 picofrit column (100 µm internal diameter. 5 µm bead size Nikkyo Technos. Tokyo. Japan) installed on to the nano-electrospray ionization source. Solvent A was 97% water, 3% acetonitrile, 0.1% formic acid; and solvent B was 5% water,

95% acetonitrile, 0.1% formic acid. At a constant flow of 0.4 µl min^{-1} the curved gradient went from 2%B up to 40%B in 45 min. followed by a steep increase to 100%B in 5 min.

The survey scan performed in the Orbitrap with 30 000 resolution (and mass range 300-2000 m/z) was followed by data-dependent MS/MS (centroid mode) in two stages: first the top five ions from the master scan were selected for collision-induced dissociation (CID) using 35% normalized collision energy with ion trap mass spectrometry (ITMS) detection; and after the same five ions underwent higher energy collision dissociation (HCD) using 37.5% normalized collision energy (7500 resolution) with detection in the Orbitrap (FTMS). Precursors were isolated with a 2 m/z window. Automatic gain control (AGC) targets were 1 x 10^6 ions for MS and for MS/MS 3 x 10^4 (CID) and 5 x 10^4 (HCD).

Maximum injection times were 500ms for MS1 and for MS2 500ms (HCD) and 200 ms (CID). The entire duty cycle lasted ~3.5s. Dynamic exclusion was used with 90s duration. Precursors with unassigned charge state or charge state 1 were excluded.

Peptide and protein identification

All Orbitrap data was searched using Sequest-Perculator under the software platform Proteome Discoverer 1.3 (Thermo) against human Ensembl database (release 75) and filtered to a 1% FDR cut off. A precursor mass tolerance of 10 ppm and product mass tolerances of 0.02 Da for HCD-FTMS and 0.8 Da for CID-ITMS were used. Further settings used were: trypsin with 1 missed cleavage; carbamidomethyl on cysteine. iTRAQ 8-plex on lysine and N-terminus as fixed modifications, oxidation of methionine and for phosphorylation searches phosphorylation of serine, threonine and tyrosine as variable modifications. Quantification of reporter ions was done by Proteome Discoverer on HCD-FTMS tandem mass spectra using an integration window tolerance of 20ppm. Only unique peptides in the data set were used for quantification.
