# Supplementary material for: Two subgroups in systemic lupus erythematosus with features of antiphospholipid or Sjögren’s syndrome differ in molecular signatures and treatment perspectives
Source: Arthritis Res Ther. 2019 Feb 18;21:62. doi: 10.1186/s13075-019-1836-8 (PMC6378708; doi:10.1186/s13075-019-1836-8)
Supplement: Supplementary file 6 — Table S3. The correlation between RF-IgM and the 25 proteins in Table 2 are shown. (PDF 256 kb) [file 13075_2019_1836_MOESM6_ESM.pdf]

Detailed Method Description: Affinity-based Proteomics

Creating the suspension bead array.

Antibodies from the Human Protein Atlas (HPA) were utilized in the screening of candidate markers using suspension bead arrays [1]. The bead array and plasma profiling assays were run as previously described [2, 3]. In brief, each antibody were diluted in MES buffer (0.1 M 2(N-Morpholino)ethanesulfonic acid, pH 5.0, Sigma-Aldrich, St. Louis, MO, USA) to 17.5 µg/ml. Then $5 \cdot 10^5$ carboxylated color-coded magnetic beads (MagPlex, Luminex Corp., Austin, TX, USA) per ID were activated by a solution of 5 µg/mL 1-ethyl-3-[3-dimethylaminopropyl]carbodiimide hydro- chloride (Thermo Scientific Pierce) and 5 µg/mL N-hydroxysulfosuccinimide (Thermo Scientific Pierce) in 100 µl monobasic sodium phosphate (0.1 M Sigma, pH 6.2) in 20 min on shaker in dark. Then the beads were washed 2x100 µl in MES buffer and then 100 µl of each antibody diluted in MES buffer were added to the beads and incubated for 2h on shaker. Finally, the beads were washed with 2x100 µl PBS-T (0.05% (v/v) Tween-20) following storage in 50µl blocking protein solution (Blocking reagent for ELISA, Roche) of 27 mg/ml supplemented with 0.1% (v/v) preservative (Proclin 300, Sigma) and stored at 4°C until usage.

Biotinylation of samples

Plasma samples were thawed overnight in 4°C, vortexed and centrifuged for 2 min at 2,000 x g, and then 3 µl of each sample was diluted in 22 µl PBS by using liquid handler (CyBi-SELMA, CyBio) followed by addition of 5 µl 10 mg/ml N-hydroxysulfosuccinimid-polyethylene oxide biotin (NHS-PEO4-Biotin, Pierce) dissolved in dimethyl sulfoxide (DMSO, Sigma) and incubated at 4°C for 2h with vortexing. Finally adding 12.5 µl of 1M Tris-HCL (pH 8.0) to each well to quench the biotinylation, and storing the samples in -20°C until usage.

Assay

Biotinylated samples were thawed in 4°C, vortexted and centrifuged for 1 min at 2,000 x g, before diluted 1:50 in a PBS buffer containing 0.1 % (w/v) casein, 0.5 % (w/v) polyvinylalcohol, 0.8 % (w/v) polyvinylpyrrolidone (all Sigma) and 0.5 mg/ml nonspecific rabbit IgG (Bethyl), with liquid handler (CyBi-SELMA, CyBio). The samples were then heat treated for 30 min at 56°C and then cooled to RT for 15 min using a water bath (TW8, Julabo). The beads were pooled and adjusted so that 5 µl contained 100 beads of each bead ID, and then 5 µl was combined with 45 µl sample and transferred to a microtiter plate (flat bottom half area, Greiner Bio-One) by using a liquid handler (CyBi-SELMA, CyBio) and incubated overnight on shaker at RT. Beads were then washed using a plate washer (EL406, Biotek) in 3 x 60 µl PBS-T (0.05 % (v/v)), and then incubated on shaker at RT with 50 µl fixing solution of 0.4% paraformaldehyde diluted in PBS for 10 min. The beads were then washed 3 x 60 µl PBS-T with a plate washer before 50 µl of 0.5 µg/ml streptavidin-conjugated R-phycoerythrin (Invitrogen) in PBS-T was added, followed by an incubation on a shaker for 20 min at RT. Beads were then washed 3x60 µl with PBS-T, followed by a final addition of 60 µl PBS-T before the fluorescent signals were measured using a FlexMap3D instrument (Luminex Corp.)

Data analysis

The measured signals reported as median fluorescent intensities (MFI) from FlexMap3D were exported into R, a program for statistical computing and graphics [4]. Outliers were identified by robust principal component analysis (R package: rrcov) and excluded from further analysis, and Normalization was based on the MFIs minimizing batch effects on MA coordinates as previously described [5]. In total, three SSA/SSB+ samples and four APL+ samples were excluded from the affinity-based proteomics analysis. As a negative control, an isotype control

of rabbit IgG was coupled to beads, and only analytes with at least two times the standard deviation plus the median of the negative control for at least one of the sample groups were kept. Group-wise analysis between the controls, aPL+ subgroup and SSA/SSB+ subgroup were done by performing Wilcoxon rank sum test selecting the 25 most significant antibodies after Bonferroni correction for multiple testing (p-value of <0.01).

1. Nilsson P, Paavilainen L, Larsson K, Odling J, Sundberg M, Andersson A-C, Kampf C, Persson A, Al-Khalili Szigyarto C, Ottosson J *et al*: **Towards a human proteome atlas: high-throughput generation of mono-specific antibodies for tissue profiling**. *Proteomics* 2005, **5**(17):4327.
2. Drobin K, Nilsson P, Schwenk JM: **Highly multiplexed antibody suspension bead arrays for plasma protein profiling**. *Methods in molecular biology (Clifton, NJ)* 2013, **1023**:137.
3. Ayoglu B, Haggmark A, Khademi M, Olsson T, Uhlen M, Schwenk JM, Nilsson P: **Autoantibody profiling in multiple sclerosis using arrays of human protein fragments**. *Mol Cell Proteomics* 2013, **12**(9):2657-2672.
4. Ihaka R, Gentleman R: **R: A Language for Data Analysis and Graphics**. *Journal of Computational and Graphical Statistics* 1996, **5**(3):299-314.
5. Hong M-G, Lee W, Nilsson P, Pawitan Y, Schwenk JM: **Multidimensional Normalization to Minimize Plate Effects of Suspension Bead Array Data**. *Journal of Proteome Research* 2016, **15**(10):3473-3480.
